# Spiritual Needs of Elderly Living in Residential/Nursing Homes

**DOI:** 10.1155/2013/913247

**Published:** 2013-08-21

**Authors:** Nora-Beata Erichsen, Arndt Büssing

**Affiliations:** ^1^Quality of Life, Spirituality and Coping, Institute of Integrative Medicine, Witten/Herdecke University, 58313 Herdecke, Germany; ^2^Freiburg Institute for Advanced Studies (FRIAS), Universität Freiburg, 79104 Freiburg, Germany

## Abstract

While the research on spiritual needs of patients with chronic and life-threatening diseases increases, there is limited knowledge about psychosocial and spiritual needs of elderly living in residential/nursing homes. We were interested in which needs were of relevance at all, and how these needs are related to life satisfaction and mood states. For that purpose we enrolled 100 elderly living in residential/nursing homes (mean age 84 ± 7 years, 82% women) and provided standardized questionnaires, that is, Spiritual Needs Questionnaire (SpNQ), Brief Multidimensional Life Satisfaction Scale (BMLSS), Quality of Life in Elders with Multimorbidity (FLQM) questionnaire, and a mood states scale (ASTS). Religious needs and Existential needs were of low relevance, while inner peace needs were of some and needs for giving/generativity of highest relevance. Regression analyses revealed that the specific needs were predicted best by religious trust and mood states, particularly tiredness. However, life satisfaction and quality of life were not among the significant predictors. Most had the intention to connect with those who will remember them, although they fear that there is limited interest in their concerns. It remains an open issue how these unmet needs can be adequately supported.

## 1. Introduction

In societies with an increasing number of elderly which are not able to care for themselves any longer and thus decide—or others may have decided for them—to live in protected housing estates (i.e., residential homes, assisted accommodation, or residential nursing homes), there is a need to care not only for their physical health but also for their psychosocial aspects. Very old individuals living alone are often depressed; the risk factors include living in distance from family and low satisfaction with living accommodation and finances [1]. However, individuals may experience depression also in nursing homes; the risk factors involve physical affections and limitations, loneliness and lack of social support, and so forth [2]. Golden et al. [3] clearly showed that loneliness and social networks have an independent influence on mood and well-being of community-dwelling elderly. In their study enrolling 1,299 elderly, 35% described themselves as lonely and 34% had a nonintegrated social network; nevertheless, also 32% of participants with an integrated social network reported being lonely [3]. There is an obviously complex network of influencing variables which all point to the fact that older individuals require psychosocial support which is not available to their satisfaction. 

A study from the late 80th stated that “older persons have significant needs that cannot be met by psychotherapy, social work, or other disciplines,” particularly because these elderly “often feel useless and without dignity” on the one hand and have to struggle with “thoughts of dying” on the other hand [4]. A small pilot enrolling ten patients from a care of the elderly assessment unit found that elderly patients stated needs “related to religion, meaning, love and belonging, morality, and death and dying” [5]. 

Such needs indicate a gap between specific expectations and the situation as it is, or, in other words, “if the individual resources to deal with the challenges (*⋯*) are insufficient to restore well-being, patients may express specific needs” [6]. With respect to patients' unmet needs, patients may expect that the fulfillment of their spiritual needs can have a positive influence on their quality of life and life satisfaction. Of course expectations can be higher as they can be fulfilled, and the respective needs remain idealistic intentions, yet this will not argue against the fact that individuals may express such needs because they regard them as important for their current situation [6, 7]. 

Spiritual needs do not necessarily refer to religious issues only, and they are not exclusively existential, too. From a theoretical point of view it is appropriate to differentiate psychosocial, existential and religious needs, yet, it is not practicable to separate these interconnected needs in a clinical context. Moreover, a specific need may have a religious connotation for one individual, and may have a clear existential connotation for an a-religious person. Moreover, the interpretation whether or not a specific need is a “spiritual” one depends on the individual attitudes and convictions, the underlying world view and the specific cultural context. 

Our recent framework of spiritual needs for research and clinical practice thus distinguishes between four interconnected core dimensions of spiritual needs [8], that is, Connection, Peace, Meaning/Purpose, and Transcendence, and correspond to the underlying categories social, emotional, existential, and religious. These dimensions can be related to Alderfer's ERG model [9] which includes the needs categories Existence (i.e., physiological and safety needs), Relatedness (i.e., belongingness and external esteem needs), and Growth (i.e., self-actualization and internal esteem needs) (see [9]). According to the ERG model, specific needs may have stronger relevance particularly when other needs cannot be fulfilled. For example, when needs for self-actualization and internal esteem cannot be fulfilled under a given situation, then relational needs (i.e. family, friends, and religious sources) would become more important.

However, most of the studies addressing spiritual needs refer to chronic patients' needs [6–20], and these patients are in most cases of higher age. Yet, the spiritual needs of patients with chronic and fatal diseases might be different from the needs of elderly which experience a decrease of their physical and mental abilities and an increasing social isolation, but must not necessarily be ill. So far research has verified that patients with life-threatening and/or chronic diseases regard their spirituality as a beneficial resource to cope [21–28], and thus acknowledging and supporting their spirituality are a main issue of spiritual care. But what about elderly which must not necessarily suffer from chronic illness but from increasing physical, mental and social restrictions? Do they have specific unmet spiritual needs? 

The aim of this study was thus to analyze which psychosocial and spiritual needs were reported by elderly living in residential/nursing homes, and how these needs are connected with life satisfaction and mood states. We included also related variables such as perceived daily life impairment and self care abilities on the one hand, and religious trust on the other hand. 

## 2. Materials and Methods

### 2.1. Individuals

All individuals of this anonym cross-sectional study were informed about the purpose of the study, were assured of confidentiality, and consented to participate. One-hundred elderly living in 12 different residential/nursing homes for elderly and assisted accommodation homes from Schleswig-Holstein (northern parts of Germany, predominantly with a Protestant denomination were enrolled. The respective institutions were chosen because of their willingness to participate and convenient accessibility. 

Inclusion criteria were age at least 65 years and written consent to participate; exclusion criteria were acute and significant health affections, and acute psychiatric disease which would impair the validity of obtained answers. We also did not include elderly with significant dementia. 

When possible, nursing staff was consulted to advise which individuals might be suited to participate. Due to the fact that most of the interviewed persons had problems with reading and writing, the interviewer red the respective items to them and assisted filling the respective answers. During this process, all comments which would help to interpret the data were recorded.

Most of the contacted individuals showed interest to participate. Although some of these volunteers were first skeptically reserved because they had to talk about private concerns, they responded nevertheless willingly during the interviews. We strictly followed the commitment of voluntariness, and thus none of the residents was coerced to participate. Only 20 persons were not willing to participate. 

### 2.2. Measures

#### 2.2.1. Psychosocial and Spiritual Needs

To measure psychosocial and spiritual needs, we used the Spiritual Needs Questionnaire (SpNQ) [7, 17]. This instrument can be used as a diagnostic instrument with 29 items and also as a validated measure of spiritual needs relying on 19 items [7]. The instrument differentiates 4 main factors, that is,

1. *religious needs* (Cronbach's alpha = .92), that is, praying for and with others, and, by themselves, participate at a religious ceremony, reading of spiritual/religious books, and turn to a higher presence (i.e., God, angels);
2. *existential needs* (reflection/meaning) (alpha = .82), that is, reflect previous life, talk with someone about meaning in life/suffering, dissolve open aspects in life, talk about the possibility of a life after death, and so forth;
3. need for *inner peace* (alpha = .82), that is, wish to dwell at places of quietness and peace, plunge into the beauty of nature, finding inner peace, talking with other about fears and worries, and devotion by others;
4. need for *giving/generativity* (alpha = .74) which addresses the active and autonomous intention to solace someone, to pass own life experiences to others, and to be assured that life was meaningful and of value.

For this analysis, we used three additional items asking for the need to be “more involved by the family in their life concerns,” to be “invited (again) to private meetings with friends,” and to “receive more support from the family.” 

All items were scored with respect to the self-ascribed importance on a 4-point scale from disagreement to agreement (0—not at all; 1—somewhat; 2—very; and 3—extremely).

#### 2.2.2. Religious Trust

To analyze religious trust, which should be associated with *religious needs* but not necessarily with the other needs, we used the respective 5-item subscale of the SpREUK questionnaire (SpREUK is an acronym of the German translation of “Spiritual and Religious Attitudes in Dealing with Illness”) [29, 30]. The scale avoids exclusive terms such as God, Jesus, or church in order not to exclude any and thus is suited particularly to secular societies. The Trust scale (alpha = .91), or trust in higher guidance/source, is a measure of intrinsic religiosity and deals with trust in spiritual guidance in life, the feeling to be connected with a higher source, trust in a higher power which carries through whatever may happen, and conviction that death is not an end, and so forth.

The scale scores items on a 5-point scale from disagreement to agreement (0, does not apply at all; 1, does not truly apply; 2, do not know (neither yes nor no); 3, applies quite a bit; and 4, applies very much). For all analyses, we used the mean score which was referred to a 100% level (transformed scale score). Scores >50% indicate higher agreement (high Trust), while scores <50% indicate disagreement (low Trust).

#### 2.2.3. Life Satisfaction

Life satisfaction was measured using the Brief Multidimensional Life Satisfaction Scale (BMLSS; alpha = .87) [31]. The items address intrinsic (myself, life in general), social (friendships, family life), external (work situation, where I live), prospective dimensions (financial situation, future prospects) of life satisfaction, and also satisfaction with the abilities to manage daily life concerns and satisfaction with the health situation. In this study, we did not measure satisfaction with work situation. 

Each of these 9 items was introduced by the phrase “I would describe my level of satisfaction as …” and scored on a 7-point scale ranging from dissatisfaction to satisfaction (0—terrible; 1—unhappy; 2—mostly dissatisfied; 3—mixed (about equally satisfied and dissatisfied); 4—mostly satisfied; 5—pleased; and 6—delighted). The BMLSS sum scores were referred to a 100% level (“delighted”). Scores >50% indicate higher life satisfaction, while scores <50% indicate dissatisfaction.

#### 2.2.4. Quality of Life in Elders with Multimorbidity

While the BMLSS measures satisfaction with defined aspects of life satisfaction, the FLQM (FLQM, acronym of the German translation “quality of life in elders with multimorbidity”) focuses on self-ascribed domains of utmost individual importance [32, 33]. Participants were invited to designate five to seven domains which are important to provide meaning, satisfaction, and well-being in their life. Then each of these dimensions is scored on a 6-point scale to assess both satisfaction (1 = there is hardly anything to improve; 2 = very satisfied; 3 = satisfied; 4 = rather unsatisfied; 5 = very unsatisfied; and 6 = it could hardly be worse) and relative importance (1 = among the most important things in my life; 2 = very important; 3 = important; 4 = somewhat important; 5 = rather unimportant; and 6 = not important compared to the other domains). For final overall scoring, both scores were inversely recoded to indicate favorable outcome by higher values. A global life satisfaction sum score (FLQM Index) is calculated (domain-specific satisfaction ∗ domain-specific weight), corrected by the individual sum of domain-specific weights. Lower scores indicate lower life satisfaction/quality of life (theoretical range 1–6).

#### 2.2.5. Mood States

To assess mood states which are related to life satisfaction and subjective quality of life, we relied on the ASTS scale (“Aktuelle Stimmungslage”) of Dalbert [34] which refers to the Profile of Mood States (POMS) [35]. It measures the state component of subjective well-being and differentiates five mood states, that is, positive mood (6 items), sorrow (3 items), despair (3 items), and tiredness (4 items). The internal consistency of the factors ranges from alpha = .83 to .94. The scale has a 7-point rating scale ranging from 0 (not at all) to 7 (very strong). Dalbert [34] has provided evidence for the external validity of the ASTS. Currently there are no norm values for the respective scales.

#### 2.2.6. Self-Perceived Daily Life Affections

Self-perceived impairment of daily life (i.e., affected by pain, ability to walk, illness, etc.) was measured with a visual analogue scale (VAS) ranging from 0 (none) to 100 (unbearable). 

### 2.3. Statistics

Descriptive statistics as well as analyses of variance, first-order correlations, and regression analyses were computed with the SPSS 20.0 software. We judged a *P* < .05 as significant; for correlation analyses we chose a significance level *P* < .01. With respect to classifying the strength of the observed correlations, we regarded *r* > .5 as a strong correlation, an *r* between .3 and .5 as a moderate correlation, an *r* between .2 and .3 as a weak correlation, and *r* < .2 as no or a negligible correlation.

## 3. Results

### 3.1. Description of the Sample

As shown in Table 1, 82% of enrolled persons were female and 18% male. Their mean age was 84 ± 7 years. Although most had a Christian denomination (84%), 56% would not describe themselves as religious; thus, also the religious Trust scores were low in the sample (Table 1).

Most were living alone but had access to their family (Table 1). They were recruited in residential homes (73%) and assisted accommodation homes (20%), while only 7% were recruited in nursing homes (mainly due to the relatively large amount of individuals with dementia which were not included). When asked for their self-care abilities (in terms of washing, dressing, mobility, etc.), 28% were independent and 27% partially independent, while 45% were completely depending on external help (Table 1).

Self-assessed daily life affections scored moderately indicating that they feel “somewhat” to “moderately” affected. Life satisfaction scored on both scales (BMLSS an FSQM) in the moderate upper range indicating that they were mostly satisfied with their life (Table 1). 

### 3.2. General Comments

During the assisted interviews, several started to weep (both women and men) because they were never confronted directly with their inmost perceptions, and they never were invited to talk about these perceptions and needs. Often the interviewees regarded these talks as “liberating,” pleasant, and enriching. 

Of importance for the interpretation of the data was the fact that several regarded the term “needs” as too strong and replaced it with the term “wish.”

Because most of the elderly were raised with a specific religious background, several were skeptic using the term “spirituality” because it was misinterpreted as esoteric and occultism on the one hand and engrossed religiosity and abstract transcendent convictions on the other hand. The term “believing” instead of “religious” was preferred because several intended to distance themselves from the church as an institution. 

Several argued that they never have had spiritual needs or some kind of faith or that they have lost their faith because of their experiences during World War II or “strokes of fate,” while for others drastic experiences confirmed their faith or they developed distinct forms of wide and trustful philosophies. 

For the following description of specific needs we added individual statements as comments or interpretations of the respective findings.

### 3.3. Ranking of Specific Spiritual Needs

The ranking of the specific needs showed a wide range of relevant needs (Table 2). The need to “plunge into beauty of nature” was the strongest, followed by the needs to “feel connected with family” and to be “invited again by friends,” to “reflect previous life,” to be “complete and safe,” to “turn to someone in a loving attitude,” to “solace someone,” and so forth. Although the interviewees' comments indicate that most feel connected with their family, they nevertheless fear to burden their family with their own troubles, fears, and worries, and thus the wish to “receive more support from the family” and to be “reinvolved by the family in their life concerns” was relatively low. This clearly contrasts with the stated needs to “turn to someone in a loving attitude” and to “give away something” (Table 2) which refers to the own children and grandchildren. 

#### 3.3.1. Talking with Others

Talking with someone about “life after death,” about “meaning in life,” or “meaning in illness and/or suffering” was of low relevance with respect to the scoring, while talking about “fears and worries” was of moderate interest (Table 2). Although family members were the preferred partners, several fear that they could burden them with their own hardships (“The children have their own life and their own worries”). Moreover, the individual statements made clear that closer relations or confiding talks with other residents were rare; often they felt an impersonal, cool, and egoistic atmosphere among the residents. Complicating was the fact that relations to close friends are reduced because several of them are already deceased or were unable to come to visits (due to decreasing physical strength or own illness). 

#### 3.3.2. Religious Issues

With respect to religious issues, it was striking that private praying was of higher relevance than praying with someone or the intention that someone is praying for the person and that “someone of your community cares for you” (Table 2). This preferred private prayer is often regarded as a “ritual” and a matter of “tradition” and refers to a few words of gratitude or the request for protection/shelter. This fits to the relatively low intention to “turn to a higher presence (i.e., God)” (Table 2).

### 3.4. Expression of Specific Spiritual Needs and Sociodemographic Variables

Relying on the respective factors of spiritual needs, it was obvious that *religious needs* and *existential needs* scored low, while *inner peace* needs scored somewhat higher and needs for *giving/generativity* the highest (Table 3). *Religious needs* were lowest in male persons, and in trend higher in those with low self-care abilities. Those with a Christian denomination had higher *religious needs* than those without any denomination (mean score: 0.6 ± 0.6 versus 0.2 ± 0.2; *F* = 8.4, *P* = .005), while all other needs did not differ with respect to denomination (data not shown). The level of education had no significant influence on the expression of SpNQ scores (data not shown). 

### 3.5. Correlations between Spiritual Needs and Quality of Life/Life Satisfaction

*Religious needs* were strongly correlated with religious trust, and moderately with the other needs scales (Table 4). *Inner Peace* needs were strongly related to *existential needs* and also *giving/generativity.*

Both life satisfaction measures (FLQM and BMLSS) were weakly (negative) associated only with needs for *Inner Peace*, while the perception of daily life affections was positively associated with *giving/generativity* but with none of the other needs scales (Table 4). 

With respect to positive and negative mood states, there were no significant (with *P* < .01) associations with positive mood or despair, while grief was moderately associated with *inner peace* needs (Table 4). Detail analyses revealed that grief was particularly associated with the need to “talk with others about fears and worries” (*r* = .41), to “find inner peace” (*r* = .39) and to “receive more attention” (*r* = .37). In contrast, tiredness was moderately correlated with *existential needs*, *inner peace* needs, and weakly also with *religious needs* and *giving/generativity*. Detail analyses showed that tiredness was particularly associated with the need to “talk with others about fears and worries” (*r* = .42), to “pray with someone” (*r* = .34), and to “find meaning in illness and/or suffering” (*r* = .34).

### 3.6. Predictors of Spiritual Needs

Because we empirically investigated several variables that could have influenced the spiritual needs, we performed regression analyses to identify the most significant predictors (Table 5). The variables which were recognized to have a significant impact on the respective needs included the aforementioned significant variables, that is, mood states, life satisfaction, daily life affections, religious Trust, and also self-care abilities. As shown in Table 5, *religious needs* can be predicted best (*R* ^2^ = .67) by religious trust, followed by tiredness and negatively by age. *Existential needs* were predicted with lower power (*R* ^2^ = .25) by tiredness and, however, by positive mood. *Inner peace* needs were predicted (*R* ^2^ = .37) best by religious trust, and grief, tiredness and negatively by age. Needs for* giving/generativity *were predicted with low power (*R* ^2^ = .20) by religious trust and tiredness. In all regression models, life satisfaction was not among the significant predictors. Since the regression coefficients may be compromised by collinearity, we checked the Variance Inflation Factor (VIF) as an indicator for collinearity. A VIF higher than 10 is indicative of high collinearity. In all cases, the Variance Inflation Factor was <2.5 indicating that collinearity was not present in the respective models. 

## 4. Discussion

### 4.1. Interpretation of Needs

We investigated psychosocial and spiritual needs of elderly living in retirement/nursing homes and found that most needs scored low when compared to the responses of patients with chronic pain diseases or cancer [6]. The wish to “plunge into beauty of nature” was expressed highest, also the needs to “feel connected with family,” to “turn to someone in a loving attitude” and to “reflect previous life” were of strong relevance. All these needs or wishes can be interpreted as the intention to (re-)connect-with environment/nature, with others, and with own life. Also the intention to “pass own life experiences to others,” to “give away something,” and to “solace someone” can be seen in this light. Obviously the residents have something to offer; they would like to connect with those who will remember them. This motif points to Erikson's psychosocial stage of development called “generativity” [36], which refers to the ability to care for others, guide the next generation, and to be assured that the own life was meaningful to others. However, although the residents may have this wish or intention, it seems that they have made the experience that there is limited interest in their offer. 

In line with this was the finding that the needs to talk with others, which is a domain of relatedness with respect to Alderfer's ERG model [9], may depend on the topic. Talking with someone about “life after death,” “meaning in life,” or “meaning in illness and/or suffering” was of lower relevance than talking about “fears and worries.” Again one may refer to individual statements that the residents may fear to burden others with their concerns. It seems that they are reluctant to talk about suffering, death and valediction; instead they intend to talk about “fears and worries”, yet they lack closer relations which would facilitate this (because several of their friends are already dead or too ill to visit them). This topic requires further analyses in future studies. 

Of interest was the fact that only 35% of the residents regarded themselves as religious (and, thus, religious trust scored low). In line with this, residents' *religious needs* were of minor relevance—with the exception of praying. It might be that praying is much more a ritual to them than an indicator of a specific longing for higher support, as verified by their limited interest to “turn to a higher presence”. Although connection and relatedness are important topics in this population, it is obvious that this intention to relate does not refer to “someone of the community” (i.e., priest, chaplain) or to “praying with someone” or that “someone is praying” for them. Although organized forms of religiosity seem to be less important, participation at a religious ceremony (i.e., service attendance) was nevertheless a need of relevance. Similar to their intention to pray (i.e., to express gratitude or to request for protection/shelter) one may suggest that this participation at religious ceremony is much more a matter of tradition which would connect them to their childhood experiences, and could also provide some kind of consolidation. 

With respect to Alderfer's ERG model [9] it seems that, for the elderly living in retirement/nursing homes, where the existence dimension may become more and more insecure, and the possibilities to develop (in terms of growth needs) are restricted, their needs for relatedness (with friends and family) have become of outstanding relevance. Interestingly, this relatedness refers to concrete others rather than transcendent sources in terms of a religion.

### 4.2. Associations between Spiritual Needs and Life Satisfaction Associated Variables

Borg et al. [37] investigated people aged 65 to 89 living in six European countries and found that their life satisfaction was significantly associated with overall health, self-esteem and negative mood such as worry, rather than reduced activities of daily living. In our study, in all regression models life satisfaction was not among the significant predictors, instead mood states. Correlation analyses revealed that needs for *Inner Peace* were influenced by negative mood states such as grief and tiredness, and by low life satisfaction; also *Existential Needs* were moderately related to tiredness. Thus, in elderly negative mood states (which are negatively related to life satisfaction, i.e., grief *r* = −.53, and tiredness *r* = −.30) seem to be one of the contributors to report specific spiritual needs. Nevertheless, the best predictors of elderlys' various spiritual needs were religious trust and mood states, particularly tiredness. *Inner Peace* needs were predicted best by religious trust, and by grief and tiredness. One may argue that these *Inner Peace* needs may simply mean to find rest and peace in the own residence (in terms of a sanctuary when nothing more is left) or that they may have a religious connotation (i.e., salvation, resurrection after death), and thus the experience of grief and tiredness might be associated with the hope that there might be “comfort” by a transcendent source (“heaven”). This suggestion would be underlined by the fact that also *Religious needs* and *Giving/Generativity *were predicted by religious trust and tiredness; it may refer to a “tiredness of life” rather than a lack of sleep, and thus the own religiosity could be a source to provide a “secure haven”. However, these hypotheses have to be verified in future studies. The predictor pattern of *Existential needs* was surprising because these needs were predicted best (albeit with weak predictive power) by tiredness and also by positive mood. Both are only weakly related (*r* = −.20), and thus more complex explanations have to be suggested.

## 5. Limitations

A limitation of this study was the cross-sectional design, which does not allow for causal interpretations; longitudinal studies are needed to substantiate the findings of this study. Moreover, the data might not be representative for all elderly living in retirement homes (particularly men are underrepresented). One may suggest that not only regional differences but also differences in the commitment of the retirement/nursing home operators may have an influence whether the residents feel adequately supported or not. Further studies which enroll retirement home residents from different areas and operators are currently in preparation. 

## 6. Conclusion and Outlook

Elderly living in residential and nursing homes have specific psychosocial and spiritual needs which are in most cases not recognized and can thus not be addressed. As advised by Borg et al. [37] adequate health care for elderly should not only consider decreasing functional capacities of elderly, but also the individual's perception of health and self-esteem. The focus on personal factors seems to be of outstanding importance, and the findings of this study support this recommendation. However, it remains an open issue how these factors can be adequately supported. 

Shea [4] advised that pastoral care specialists might be beneficial because they could help finding “inner power that produces hope and character”. However, most of the enrolled elderly had no specific interest in priests or chaplains. Other professions such as nurses, psychologists and social workers might be in charge to care, to listen and to help elderly to review their life which was a strong need among the residents enrolled in this study. Of course they might not be the most appropriate partners (the family seems to be of stronger importance), but they are often those who are more easily available, and they may be able to identify disrupted relationships. It is obvious that there is a need to “develop creative, long-range strategies for providing care” [4] which meet the needs of elderly living in secular societies. One interesting approach could be life reviews or reminiscing interventions. A randomized study by Gonçalves et al. [38] found that a life review decreased depressive symptoms and contributed to improve older womens' life satisfaction. A further randomized controlled study reported significantly lower depressive symptoms in elderly participating an autobiographical writing workshop [39]. In contrast, a randomized controlled study by de Medeiros et al. [40] found that an autobiographical writing workshop may improve the ratings of self-concept among older adults, but not their mood when compared to the outcomes of a reminiscence group (which was an active control intervention) or to a no-treatment control group. At least such life reviews may contribute to connect elderly with their own past, and can function as a “legacy” of life experiences to connect with future generations. This will meet the specific needs to reflect previous life and to pass own life experiences to others, and thus to connect with those who will remember them. 

We hope that the current data and reflections encourage a discussion which integrative approaches might be appropriate to support elderly living in residential and nursing homes. Although one may assume that particularly interventions of the wide field of mind-body medicine might be beneficial, this remains to be verified.

## Figures and Tables

**Table 1:** Sociodemographic and psychometric data of 100 interviewed elderly.

Variables	Mean/%
Age (mean ± SD)	84 ± 7
Gender (%)	
Men	18
Women	82
Family status (%)	
Married	13
Single/divorced	19
Widowed	68
Access to family (%)	88
Education level (%)*	
Primary/secondary	55
(Junior) high school	45
Denomination (%)	
Christian	84
None	16
Self-perceived religiosity (%)	
Religious	35
Not religious	56
Undecided	9
Type of institution (%)	
Residential home	73
Assisted accommodation	20
Residential nursing home	7
Self-care abilities (%)	
Independent	28
Largely independent	27
Only with help	45
Religious trust (SpREUK; mean ± SD; range 0–100)	38 ± 29
Daily life affections (VAS; mean ± SD; range 0–100)	44 ± 27
Life satisfaction (BMLSS; mean ± SD; range 0–100)	68 ± 13
Life satisfaction (FSQM; mean ± SD; range 1–6)	4.2 ± 0.9

Results are means ± standard deviation (SD) or percentage of the respective sociodemographic or psychometric variables. *Categorization of education level has to be interpreted with caution because it refers to the conditions of the time period from 1920 to 1950 with limited access to higher education. BMLSS: Brief Multidimensional Life Satisfaction Scale; FLQM: “quality of life in elders with multimorbidity” questionnaire; SpREUK: “Spiritual and Religious Attitudes in Dealing with Illness” questionnaire; VAS: visual analogue scale.

**Table 2:** Ranking of specific needs.

Item number	Stated needs	Mean	SD	Related factor
N6	Plunge into beauty of nature	2.23	1.00	IP
N25	Feel connected with family	1.89	1.26	—
N4	Reflect previous life	1.56	1.17	EN
N13	Turn to someone in a loving attitude	1.52	1.24	IP
N24	Being complete and safe	1.38	1.29	—
N29	Invited for private meetings by friends again	1.26	1.18	—
N14	Give away something from yourself	1.25	1.24	(GG)
N15	Solace someone	1.09	1.20	GG
N20	Pray for yourself	1.01	1.10	RN
N9	Listen to touching music	0.99	1.12	—
N26	Pass own life experiences to others	0.98	1.06	GG
N21	Participate at a religious ceremony	0.94	1.10	RN
N7	Dwell at a place of quietness and peace	0.91	1.20	IP
N2	Talk with others about fears and worries	0.70	1.12	IP
N23	Turn to a higher presence	0.70	1.10	RN
N1	Receive more attention	0.69	1.11	—
N8	Find inner peace	0.67	1.11	IP
N11	Talk with someone about the question of meaning in life	0.55	1.00	EN
N28	Reinvolved by family in their life concerns	0.43	0.90	—
N10	Find meaning in illness and/or suffering	0.41	0.84	EN
N5	Dissolve open aspects of your life	0.37	0.87	(EN)
N22	Read religious/spiritual books	0.28	0.72	RN
N30	Receive more support from your family	0.28	0.75	—
N17	Be forgiven	0.26	0.72	(IP)
N16	Forgive someone from a distinct period of your life	0.23	0.68	EN
N12	Talk with someone about the possibility of life after death	0.22	0.66	EN
N18	Pray with someone	0.19	0.60	RN
N3	Someone of your community (i.e., priest, chaplain) cares for you	0.15	0.52	(RN)
N19	Someone prays for you	0.11	0.49	RN

Results are means ± standard deviation (SD) of the respective needs (range: 0–3). The items refer to the following factors: religious needs (RN), existential needs (EN), inner peace needs (IP), and giving/generativity (GG); some items are additional items which are not regarded as spiritual needs.

**Table 3:** Mean values of spiritual needs scores.

	Religious needs	Existential needs	Inner peace needs	Giving/generativity
All				
Mean	0.54	0.59	1.07	1.29
SD	0.58	0.49	0.63	0.91
Gender				
Female				
Mean	0.60	0.60	1.06	1.28
SD	0.59	0.49	0.61	0.92
Male				
Mean	0.26	0.59	1.11	1.31
SD	0.44	0.49	0.73	0.87

*F* value	**5.3**	0.0	0.1	0.0
*P* value	**.023**	n.s.	n.s.	n.s.

Self-care abilities				
Only with help				
Mean	0.68	0.66	1.03	1.27
SD	0.67	0.45	0.64	1.00
Largely independent				
Mean	0.39	0.50	0.91	1.19
SD	0.49	0.45	0.50	0.70
Independent				
Mean	0.45	0.58	1.27	1.42
SD	0.46	0.57	0.70	0.95

*F* value	2.7	0.9	2.4	0.6
*P* value	.074	n.s.	.092	n.s.

Results are means ± standard deviation (SD) of the respective scales (range: 0–3) as measured with the Spiritual Needs Questionnaire (SpNQ). We judged *P* < .05 as significant but indicated also statistically notable trends (.5 < *P* < 1.1).

**Table 4:** Correlations between spiritual needs, quality of life/life satisfaction, and mood states.

	Religious needs	Existential needs	Inner peace needs	Giving/generativity
Spiritual needs (SpNQ)				
Religious needs	1	0.304**	0.398**	0.360**
Existential needs		1	0.537**	0.450**
Inner peace needs			1	0.622**
Giving/generativity				1
Religious trust (SpREUK)	0.759**	0.111	0.286**	0.236
Life satisfaction/quality of life				
FLQM index	−0.079	−0.149	−0.294**	−0.056
BMLSS sum score	0.010	−0.213	−0.279**	−0.097
Daily life affections (VAS)	0.172	0.256	0.208	0.262**
Mood states (ASTS)				
Positive mood	0.043	0.047	−0.085	0.219
Grief	0.231	0.251	0.406**	0.118
Despair	0.174	0.236	0.223	0.125
Tiredness	0.271**	0.397**	0.361**	0.263**

**P < .01 (Pearson). ASTS: Profile of Mood States; BMLSS: Brief Multidimensional Life Satisfaction Scale; FLQM: “quality of life in elders with multimorbidity” questionnaire; SpNQ: Spiritual Needs Questionnaire; SpREUK: “Spiritual and Religious Attitudes in Dealing with Illness” questionnaire; VAS: visual analogue scale.

**Table 5:** Regression analyses with spiritual needs as dependent variables (enter method).

Model	Beta	*T*	*P*
Dependent variable: religious needs (*R* ^2^ = .67)			
(Constant)		.339	.735
Grief (ASTS)	.016	.177	.860
Despair (ASTS)	.093	1.187	.238
Tiredness (ASTS)	**.189**	**2.503**	**.014**
Positive mood (ASTS)	.070	.906	.367

Quality of life (FLQM Index)	−.061	−.676	.501
Life satisfaction (BMLSS)	.165	1.745	.085
Daily life affections (VAS)	.062	.823	.413
Self-care abilities	−.146	−2.194	.031

Religious trust (SpREUK)	**.699**	**10.868**	**.000**

Female gender	.068	1.051	.296
Age	**−.131**	**−2.041**	**.044**

Dependent variable: existential needs (*R* ^2^ = .25)			
(Constant)		−.139	.890
Grief (ASTS)	.115	.840	.403
Despair (ASTS)	.080	.667	.506
Tiredness (ASTS)	**.375**	**3.275**	**.002**
Positive mood (ASTS)	**.254**	**2.165**	**.033**

Quality of life (FLQM index)	.144	1.049	.297
Life satisfaction (BMLSS)	−.188	−1.306	.195
Daily life affections (VAS)	.030	.260	.795
Self-care abilities	−.093	−.922	.359

Religious trust (SpREUK)	.055	.564	.574

Female gender	−.082	−.840	.403
Age	.032	.329	.743

Dependent variable: inner peace needs (*R* ^2^ = .37)			
(Constant)		2.619	.010
Grief (ASTS)	**.265**	**2.116**	**.037**
Despair (ASTS)	−.021	−.192	.848
Tiredness (ASTS)	**.213**	**2.035**	**.045**
Positive mood (ASTS)	.021	.195	.846

Quality of life (FLQM Index)	.022	.174	.862
Life satisfaction (BMLSS)	−.136	−1.031	.305
Daily life affections (VAS)	.023	.221	.826
Self-care abilities	.178	1.931	.057

Religious trust (SpREUK)	**.279**	**3.129**	**.002**

Female gender	−.147	−1.643	.104
Age	**−.221**	**−2.491**	**.015**

Dependent variable: giving/generativity (*R* ^2^ = .20)			
(Constant)		1.059	.293
Grief (ASTS)	−.083	−.590	.557
Despair (ASTS)	.043	.349	.728
Tiredness (ASTS)	**.250**	**2.113**	**.037**
Positive mood (ASTS)	.212	1.744	.085

Quality of life (FLQM Index)	.075	.525	.601
Life satisfaction (BMLSS)	−.209	−1.404	.164
Daily life affections (VAS)	.073	.617	.539
Self-care abilities	.053	.505	.615

Religious trust (SpREUK)	**.238**	**2.357**	**.021**

Female gender	−.051	−.502	.617
Age	−.081	−.802	.425

Significant variables were highlighted (bold). *Because the regression coefficients may be compromised by collinearity, we checked the Variance Inflation Factor (VIF) as an indicator for collinearity. VIF > 10 is indicative of high collinearity. In all cases, the VIF was <2.5, and thus these data are not depicted here. ASTS: Profile of Mood States; BMLSS: Brief Multidimensional Life Satisfaction Scale; FLQM: “quality of life in elders with multimorbidity” questionnaire; SpNQ: Spiritual Needs Questionnaire; SpREUK: “Spiritual and Religious Attitudes in Dealing with Illness” questionnaire; VAS: visual analogue scale.
